# Pulmonary Vasodilator Therapy in Severe Pulmonary Hypertension Due to Chronic Obstructive Pulmonary Disease (Severe PH-COPD): A Systematic Review and Meta-Analysis

**DOI:** 10.3390/jcdd10120498

**Published:** 2023-12-16

**Authors:** Ahmed Elkhapery, M. Bakri Hammami, Roxana Sulica, Hemanth Boppana, Zeinab Abdalla, Charoo Iyer, Hazem Taifour, Chengu Niu, Himanshu Deshwal

**Affiliations:** 1 Department of Internal Medicine, Rochester General Hospital, Rochester, NY 14621, USA; ahmed.elkhapery@rochesterregional.org (A.E.); hemanthkrishna.boppana@rochesterregional.org (H.B.); charoo.iyer@rochesterregional.org (C.I.); chengu.niu@rochesterregional.org (C.N.); 2 Department of Internal Medicine, Jacobi Medical Center-Albert Einstein College of Medicine, New York, NY 10461, USA; hammamim@nychhc.org; 3 Division of Pulmonary, Sleep and Critical Care Medicine, Department of Medicine, New York University Grossman School of Medicine and NYU Langone Health, New York, NY 10016, USA; rsulica@yahoo.com; 4 Rochester General Hospital Research Institute, Rochester, NY 14621, USA; zeinab.abdallah@rochesterregional.org; 5 Department of Internal Medicine, Unity Hospital, Rochester, NY 14626, USA; hazem.taifour@rochesterregional.org; 6 Division of Pulmonary, Sleep and Critical Care Medicine, Department of Medicine, West Virginia University School of Medicine, Morgantown, WV 26505, USA

**Keywords:** COPD, severe PH-COPD, pulmonary hypertension, phosphodiesterase 5 inhibitors, prostacyclin analogs, endothelin receptor antagonists

## Abstract

**Background:** Chronic obstructive pulmonary disease-associated pulmonary hypertension (PH-COPD) results in a significant impact on symptoms, quality of life, and survival. There is scant and conflicting evidence about the use of pulmonary hypertension (PH) specific therapy in patients with PH-COPD. **Study Design and Methods:** PubMed, OVID, CINAHL, Cochrane, Embase, and Web of Science were searched using various MESH terms to identify randomized controlled trials (RCTs) or observational studies investigating PH-specific therapies in patients with severe PH-COPD, defined by mean pulmonary artery pressure (mPAP) of more than 35 mm Hg or pulmonary vascular resistance (PVR) of more than 5 woods units on right heart catheterization. The primary outcome was a change in mPAP and PVR. Secondary outcomes were changes in six-minute walk distance (6MWD), changes in the brain-natriuretic peptide (BNP), New York Heart Association (NYHA) functional class, oxygenation, and survival. **Results:** Thirteen studies satisfied the inclusion criteria, including a total of 328 patients with severe PH-COPD. Out of these, 308 patients received some type of specific therapy for PH. There was a significant reduction in mPAP (mean difference (MD) −3.68, 95% CI [−2.03, −5.32], *p* < 0.0001) and PVR (MD −1.40 Wood units, 95% CI [−1.97, −0.82], *p* < 0.00001). There was a significant increase in the cardiac index as well (MD 0.26 L/min/m^2^, 95% CI [0.14, 0.39], *p* < 0.0001). There were fewer patients who had NYHA class III/lV symptoms, with an odds ratio of 0.55 (95% CI [0.30, 1.01], *p* = 0.05). There was no significant difference in the 6MWD (12.62 m, 95% CI [−8.55, 33.79], *p* = 0.24), PaO_2_ (MD −2.20 mm Hg, 95% CI [−4.62, 0.22], *p* = 0.08), or BNP or NT-proBNP therapy (MD −0.15, 95% CI [−0.46, 0.17], *p* = 0.36). **Conclusion:** The use of PH-specific therapies in severe PH-COPD resulted in a significant reduction in mPAP and PVR and increased CI, with fewer patients remaining in NYHA functional class III/IV. However, no significant difference in the 6MWD, biomarkers of right ventricular dysfunction, or oxygenation was identified, demonstrating a lack of hypoxemia worsening with treatment. Further studies are needed to investigate the use of PH medications in patients with severe PH-COPD.

## 1. Background

Pulmonary hypertension due to chronic obstructive pulmonary disease (PH-COPD) is precapillary pulmonary hypertension (PH) classified under World Health Organization (WHO) Classification Group 3 and is associated with significant limitations in functional status, quality of life, and survival [1]. Recently, the hemodynamic definition of precapillary PH was revised as a mean pulmonary artery pressure (mPAP) of greater than 20 mm Hg at rest, pulmonary vascular resistance (PVR) of greater than 2 Wood units, and a pulmonary artery wedge pressure (PAWP) of less than or equal to 15 mm Hg [1].

Pulmonary hypertension in the setting of lung disease, such as COPD (Group 3 PH) involves vascular remodeling due to the combined effects of hypoxia, mechanical/oxidative stress, and inflammatory reactions [2,3,4]. Right heart failure secondary to pulmonary hypertension carries significant mortality, with one study demonstrating a mortality rate of 14% during hospital admission and 13%, 26%, and 35% at 3, 6, and 12 months, respectively [5]. PH can be categorized as nonsevere or severe based on hemodynamics. The 2015 European Society of Cardiology (ESC) and European Respiratory Society (ERS) Guidelines defined severe PH by mPAP > 35 mmHg or mPAP ≥ 25 mmHg, with CI < 2.5 L/min/m^2^ [6]. However, this was updated in the 2022 guidelines to use a PVR threshold of >5 WU. This change was based on studies by Zeder et al. and Olsson et al. in 2021, demonstrating a PVR threshold of >5 WU as a better predictor of prognosis in patients with COPD and ILD [7,8]. Severe PH occurs in 1–5% of patients with COPD and is associated with worse symptoms and survival; in contrast, any severity of PH in ILD is associated with increased mortality [9,10,11,12]. Patients with severe PH-COPD may have predominantly circulatory limitations to their symptoms with signs of right ventricular failure and are phenotypically distinct from COPD without PH or mild PH. The hypoxia-inducible factor-2 is considered a possible molecular switch that may result in airway versus vascular remodeling [13].

Pulmonary vasoactive medications have recently emerged at the forefront of treatment in pulmonary arterial hypertension (WHO Group 1 PH). More recently, additional evidence has supported the use of inhaled treprostinil in PH due to interstitial lung disease, with a study demonstrating improved six-minute walk test as well as reduction in N-terminal pro-BNP (NT-proBNP) levels [14]. There is also evidence for the use of PH-specific therapy in select patients with inoperable CTEPH [15]. However, there is scant evidence for their use in the treatment of PH-COPD. PH-specific therapies have been trialed in COPD with mixed results [16,17]. These studies often included both nonsevere and severe PH and used noninvasive methods (echocardiography) to estimate the pulmonary pressures. 

Currently, the use of these medications in COPD patients is limited to trial settings and compassionate use in patients with severe symptoms, with no good quality evidence available to support their use. Furthermore, there is limited evidence on the utility of PH-specific therapy in severe PH-COPD. Therefore, we performed a systematic review and meta-analysis of the available literature to investigate the use of PH-specific therapy in severe PH-COPD, defined as mPAP ≥ 35 mm Hg or PVR ≥ 5 Wood units.

## 2. Study Designs and Methods

### 2.1. Protocol and Registration

This systematic review has been registered in the PROSPERO International Prospective Register of Systematic Reviews with registration ID: CRD42023394777. The review and reporting were carried out according to the Preferred Reporting Items for Systematic Reviews and Meta-Analysis (PRISMA) guidelines.

### 2.2. Search Strategy

PubMed, OVID, CINAHL, Cochrane, Embase, and Web of Science were searched for all records to date (2 May 2023). Records in the English language were assessed for inclusion.

Search terms included (“Chronic obstructive pulmonary disease” or “COPD” or “emphysema” or “chronic bronchitis”), “pulmonary hypertension”, and (“epoprostenol” or “treprostinil” or “iloprost” or “beraprost” or “selexipag” or “sildenafil” or “tadalafil” or “udenafil” or “riociguat” or “bosentan” or “ambrisentan” or “macitentan” or “vasodilator agent” or “phosphodiesterase 5 inhibitor (PDE5i)” or “soluble guanylate cyclase simulator” or “endothelin receptor antagonist” or “prostacyclin” or “prostacyclin agonist” or “prostacyclin derivative”).

Patients were included with severe PH-COPD, defined by mPAP ≥ 35 mm Hg or PVR ≥ 5 Wood units on right heart catheterization and the exclusion of alternate etiology. 

Patients with mild or moderate pulmonary hypertension, defined as mean pulmonary artery pressure of less than 35 mmHg, were excluded. Studies on patients with ILD, or WHO Groups 1, 2, 4, or 5 PH were also excluded. Studies that did not include hemodynamic data/right heart catheterization prior to the use of the study drug were also excluded. 

### 2.3. Data Extraction

Data were extracted from the studies using a standardized and piloted data entry sheet by the first 2 authors (A.E. and M.H.) independently. Detailed data on study characteristics, including the type of study design, region, follow-up duration, and sample size, were collected. Data on baseline and follow-up mPAP, PVR, brain-natriuretic peptide (BNP), oxygenation, six-minute walk distance (6MWD), NYHA or World Health Organization (WHO) functional class, and survival (1- and 3-year mortality) were collected and analyzed. The unit of measurement was converted for uniformity (6MWD to meters, PaO_2_ to mmHg, and PVR to Wood units).

Studies that included patients with pulmonary HTN due to COPD as well as pulmonary HTN due to other causes, but reported the outcomes for each group individually, were included. Only the data about patients with pulmonary HTN due to COPD were collected and used in the meta-analysis. 

### 2.4. Statistical Analysis

Statistical analysis was performed using the Cochrane Review Manager (RevMan) version 5.3. To analyze the mean difference before and after therapy for continuous variables, the inverse variance method was used on a random-effect model. For dichotomous/categorical variables (e.g., NYHA functional class), we used the Mantel–Haenszel random-effect method to calculate the unadjusted odds ratio (OR) [18].

### 2.5. Quality of the Included Studies

We only had one randomized control trial (RCT) included in the qualitative review, by Vitulo et al. [19]. This study was deemed to have an overall low risk of bias using the ROB-2 tool [20]. Intention to treat analysis was used. There was a low risk with sequence generation. Allocation concealment was not well described in the text. Participants and personnel were blinded (double-blinded study). It was unclear if the assessor of the outcomes was blinded. The primary outcome, however, was PVR, which was invasively measured and thus unlikely to be influenced by prior knowledge of group allocation. There was a low risk of bias associated with other domains (incomplete outcome data, selective reporting, and other sources of bias).

The overall quality of the included cohort studies was moderate using the Newcastle–Ottawa Scale (NOS) [21]. Most cohort studies did not include a control group and thus lost points in the NOS quality score, as there was no selection of a nonexposed cohort and therefore no way to control for important covariates such as severity of COPD and long-term O_2_ use status. The detailed scale and scores are shown in Table 1.

## 3. Results 

### 3.1. Study Characteristics

**Figure 1:**
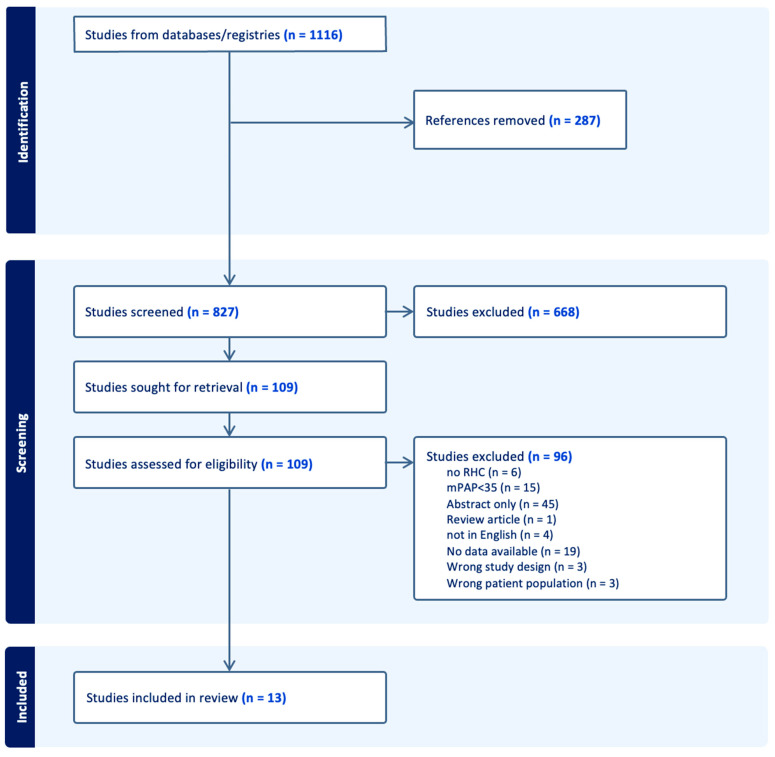
PRISMA diagram.

The initial search revealed 1116 articles. After the removal of duplicates (*n* = 287), 827 studies were screened independently by two authors (A.E and M.H.). Disagreements were solved by consensus. After the exclusion of irrelevant studies (*n* = 668), 109 articles were selected for full-text review. Of these, 96 articles were excluded based on different reasons, including insufficient data for analysis (*n* = 19), noninvasive measurement of pulmonary pressures (*n* = 6), nonsevere pulmonary hypertension (*n* = 15), abstract only (*n* = 45), and other reasons (*n* = 11). Thirteen studies satisfied the inclusion criteria in this study, including a total of 328 patients with severe PH-COPD. Out of these, 308 patients received some form of specific therapy for pulmonary hypertension. Most studies were retrospective in nature, except for two nonrandomized experimental studies by Jones et al. and Wang et al. [29,33], and a single randomized controlled trial by Vitulo et al. in 2017 [19]. The Preferred Reporting Items for Systematic Reviews and Meta-Analysis (PRISMA) flow diagram is shown in Figure 1.

There was significant heterogeneity in the type of PH-specific therapy, route, dose, and duration of therapy between the studies. Most studies had at least 3 months of therapy and follow-up, except for the studies performed by Jones et al. and Wang et al., which focused on the acute (minutes–hours) effect of the medications on pulmonary vasculature. Further details about each study are shown in Table 2.

### 3.2. Hemodynamics 

**Figure 2:**
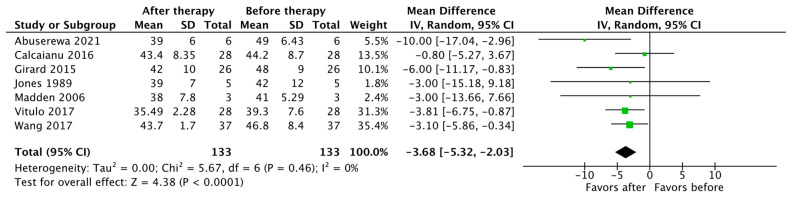
Forest plot of comparison: mean pulmonary artery pressure (mPAP) in mm Hg before vs. after PH-specific therapy [19,22,24,27,29,31,33].

**Figure 3:**
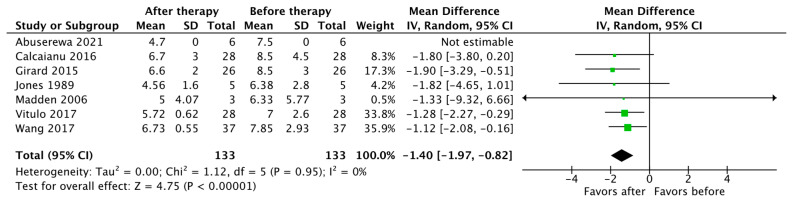
Forest plot of comparison: pulmonary vascular resistance (PVR) in Wood units [19,22,24,27,29,31,33].

Seven studies reported changes in mPAP and PVR as an outcome (*n* = 133). The pooled results show that the use of PH-specific therapy resulted in a significant reduction in mPAP, with a mean difference (MD) of −3.68 (95% CI [−5.32, −2.03], *p* < 0.0001) (Figure 2). There was also a significant reduction in PVR, with a mean difference (MD) of −1.40 Wood units, (95% CI [−1.97, −0.82], *p* < 0.00001) (Figure 3). 

**Figure 4:**
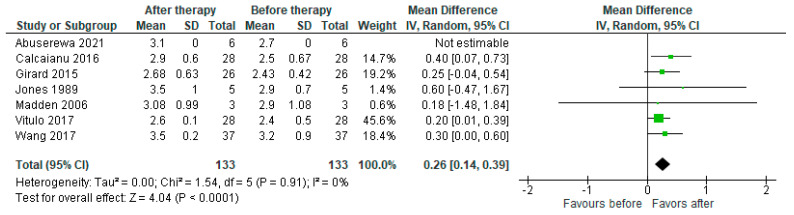
Forest plot of comparison: cardiac index (L/min/m^2^) before and after PH-specific therapy [19,22,24,27,29,31,33].

Seven studies reported a change in the cardiac index (*n* = 133), with the pooled results showing an increase in CI after therapy with a mean increase of 0.26 L/min/m^2^ (95% CI [0.14, 0.39], *p* < 0.0001) (Figure 4).

### 3.3. Exercise Capacity and Functional Status 

**Figure 5:**
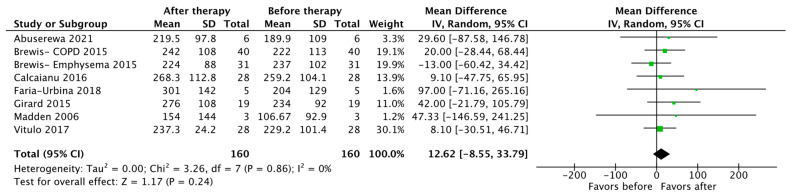
Forest plot of comparison: changes in six-minute walk distance (in meters) [19,22,23,24,25,27,31].

Seven studies reported changes in the 6MWD as an outcome (*n* = 160), with the pooled results showing no statistically significant difference after therapy (MD 12.62 m, 95% CI [−8.55, 33.79], *p* = 0.24) (Figure 5). 

**Figure 6:**
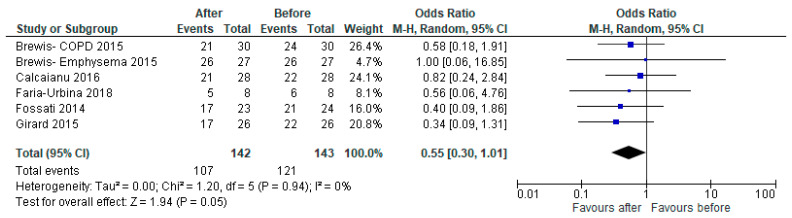
Forest plot of comparison: number of patients with NYHA or WHO functional class III or IV, before vs. after PH-specific therapy [23,24,25,26,27].

Five studies reported the NYHA or WHO functional class of patients. There were significantly fewer patients in NYHA functional class III/lV after therapy, with an odds ratio of 0.55 (95% CI [0.30, 1.01], *p* = 0.05) (Figure 6).

### 3.4. Survival and Secondary Outcomes

**Figure 7:**
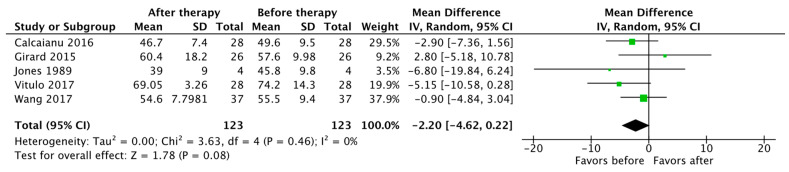
Forest plot of comparison: PaO_2_ (in mmHg) before and after PH-specific therapy [19,24,27,29,33].

Survival data were reported by five studies, with a total of 183 patients. The average 1-year and 3-year transplant-free survival of patients with severe PH-COPD on PH-specific therapy was 81% and, 41% by 3 years, respectively (Table 3). Five studies reported a change in PaO_2_ after the initiation of PH-specific therapy, with the pooled results showing no statistically significant difference (MD −2.20 mm Hg, 95% CI [−4.62, 0.22], *p* = 0.08) (Figure 7).

**Figure 8:**
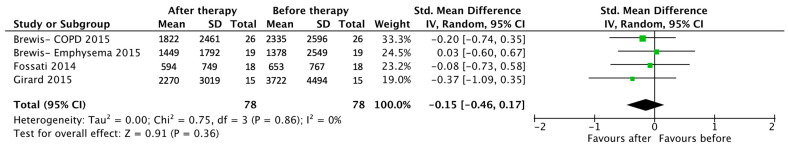
Forest plot of comparison: change in the brain-natriuretic peptide (BNP) or N-terminal pro-BNP with PH-specific therapy [23,26,27].

Changes in BNP or NT-proBNP were reported by three studies, with the pooled results showing no significant difference after the initiation of PH-specific therapy (MD −0.15, 95% CI [−0.46, 0.17], *p* = 0.36) (Figure 8).

## 4. Discussion

This systematic review and meta-analysis showed that in patients with severe PH-COPD, treatment with PH-specific therapies resulted in an overall modest reduction in PVR (MD −1.40 Wood units, 95% CI [−1.97, −0.82], *p* < 0.00001) and mPAP (MD 3.68 mm Hg, 95% CI [2.03, 5.32], *p* < 0.0001). These findings are consistent with the only randomized controlled trial we found, which was a small pilot trial studying the effect of sildenafil on severe PH associated with COPD [19]. There was a decrease of −1.4WU in PVR compared to the placebo (*p* = 0.04) at sixteen weeks, which is consistent with the findings of our review. These results are also similar to findings from a small randomized controlled trial by Blanco et al. [34], which was a dose-comparison trial in 20 patients comparing 20 mg to 40 mg of sildenafil in patients with PH-COPD, defined by mPAP > 20. The results showed that both doses of sildenafil resulted in a reduction in mPAP (−6 mm Hg, 95% CI, −7 to −4, at rest). Other studies include RCTs by Rao et al. [35] and Goudie et al. [36], showing that both sildenafil and tadalafil decrease systolic pulmonary artery pressure (sPAP), as measured with echocardiography in patients with PH-COPD. These studies, however, did not use invasive hemodynamic measurement and included patients with severe and nonsevere PH. 

In terms of exercise capacity, the results of this meta-analysis show no difference in the six-minute walk distance (6MWD) (*p* = 0.24) before and after the initiation of PH-specific therapies. However, there were fewer patients in NYHA or WHO functional class III and IV following treatment (*p* = 0.05), which may suggest that there may be a benefit in this subset of patients. The limitation in identifying a clinically meaningful impact on the six-minute walk distance may be due to the severe baseline functional limitation in patients with severe PH-COPD compared to those with nonsevere PH-COPD or Group I PH and those who have a multifactorial cause of exercise capacity impairment [37].

In the SPHERIC-1 RCT, sildenafil was associated with improved BODE (body mass index, lung obstruction, dyspnea, and exercise capacity) index, the predicted diffusion capacity of the lung for carbon monoxide (DLCO) percentage, and quality of life [19]. A study by Rao et al. (2011) showed that sildenafil resulted in an increase in the 6MWD by 190 m compared to the placebo. Although this study used echocardiography to estimate PH and did not use severe PH as a selection criterion, the baseline sPAP was 52.7 ± 11.9 mmHg. 

On the other hand, other studies show no benefit in the exercise capacity, often defined by the 6MWD [36]. A study by Blanco et al. involving 63 patients undergoing pulmonary rehab showed no significant improvement in cycle endurance gains, the incremental exercise test, 6MWD, and quality of life between patients receiving 20 mg sildenafil or the placebo three times daily [17]. However, the study included patients with a mean PAP ≥ 25 mmHg, and invasive hemodynamic measurement was not necessary for inclusion in the trial. Similarly, a small randomized controlled trial by Stolz et al. showed that the oral administration of bosentan resulted in no significant improvement in the 6MWD compared to the placebo [16]. However, this study included patients with severe COPD (symptomatic, severe, or very severe COPD and/or emphysema; GOLD classes 3–4), with echocardiography used to screen for elevated pulmonary pressure, but this was not a selection criterion. The median sPAP was 37 mmHg (IQR 20–42) in the placebo group, compared to a median of 32 mmHg (IQR 29–38) in the treatment group. This limits the generalizability of these results given the lack of invasive hemodynamic measurements and the fact patients did not have severe PH.

The COMPERA registry prospectively described the characteristics and outcomes of patients with moderate or severe PH in COPD and compared them with patients with idiopathic PAH (IPAH) [37]. This registry included 307 patients with COPD and severe PH, defined as mPAP ≥ 35 mm Hg or mPAP ≥ 25 mm Hg with CI < 2 L/min/m^2^. The results showed that patients with PH in COPD were functionally more impaired and had a poorer outcome than patients with IPAH, and transplant-free survival rates at 1, 3, and 5 years were higher in the IPAH group (94%, 75%, and 55%, respectively) than in the severe PH-COPD group (86%, 55%, and 38%; *p* = 0.004) [37]. Most patients with severe PH and COPD received at least one form of PH-specific therapy, with 93% being started on PDE-5i monotherapy within 3 months of diagnosis. Interestingly, response to therapy at 6 months was defined as an increase of ≥30 in the 6MWD or an improvement in WHO-FC. Patients with a lower 6MWD and high WHO-FC at baseline were more likely to be responders. Responders also had better transplant-free survival than nonresponders, but this observation was limited to patients with severe PH in COPD. Our study demonstrates a similar outcome of 1-year and 3-year survival in severe PH-COPD patients treated with PH-specific therapy. Unfortunately, a comparison to untreated patients could not be made due to the lack of a controlled cohort in most studies included in this meta-analysis. In addition, most studies included in our meta-analysis may not have long-term follow-up to assess true improvement in the 6MWD over time. Interestingly, our meta-analysis did demonstrate improvement in WHO-FC, with fewer patients remaining in WHO-FC III/IV.

Our study showed there was no significant reduction in PaO2 (*p* = 0.08), which some studies cited as a safety risk with the use of these therapies in patients with severe COPD. This is similar to the findings from the SPHERIC-1 RCT, which showed there was no reduction in PaO_2_ with the use of sildenafil on severe PH associated with COPD. On the other hand, a small dose-comparison RCT by Blanco et al. [34], which included COPD patients with PH defined by mPAP > 20, showed there was a reduction in PaO_2_ at rest (−6 mm Hg (95% CI, −8 to −4)), which was believed to be due to a loss of hypoxic vasoconstriction and increased perfusion in alveoli with low V/Q ratio. A small RCT showed that bosentan resulted in a drop in arterial oxygen pressure and worsened alveolar–arterial gradient [16], although this study did not involve invasive hemodynamic measurement and included nonsevere PH-COPD; thus, it was not generalizable to the severe PH-COPD phenotype. 

It is possible that the benefit seen in studies such as the SPHERIC-1 study and the study by Rao et al. is due to the higher prevalence of severe PH in these populations [19,35]. While mild PH is a relatively common feature in COPD, only 3–5% of patients with COPD develop severe PH, and the reason for this is not yet clear [10,11]. It may be due to the coexistence of PAH in patients with COPD, and it is important to rule out underlying connective tissue disease or Group I etiology in these patients. Recently, the term “pulmonary vascular phenotype” has been used to describe this subset of patients with COPD and severe PH [38,39,40], which often have less severe airflow limitation but an out-of-proportion reduction in DLCO with hypoxemia and exercise limitation that is cardiovascular in origin. Lungs explanted from patients with PH-COPD show pulmonary arterial lesions that correlate with the severity of PH and are morphologically similar to those characteristics of IPAH [41]. 

The pathogenic mechanisms that result in pulmonary vascular phenotype COPD, or “out-of-proportion” pulmonary hypertension in COPD, are not well defined, but a complex interaction between lung hyperinflation due to emphysema, increased pulmonary vascular stiffness, and hypoxic pulmonary vasoconstriction (HPV) has been suggested [42]. Vanishing capillary syndrome has also been implicated, which describes the process of lung capillary rarefaction in response to chronic hypoxic conditions, which can result in severe pulmonary hypertension with reduced DLCO in mild–moderate COPD [9,43]. Lastly, hypoxia-inducible factors (HIFs), which are transcription factors that are involved in physiologic lung development, angiogenesis, and repair, are increasingly recognized as crucial factors in the development of pulmonary hypertension [44,45]. Recent studies have implicated the upregulation of HIF-2 in the development of pulmonary vascular phenotype, with studies showing that mice overexpressing HIF-2α developed severe PH [46], while the inhibition of HIF-2α reduced the development of hypoxia-induced PH [47]. Meanwhile, studies of mice with endothelial cell (EC)-specific deletion of Hif-2α led to emphysema development [38]. This study also showed that HIF-2α overexpression was protective against the development of emphysema [48]. These observations lead to the hypothesis that HIF-2 transcription factors can control the genes that are involved in vascular remodeling and lung alveolar healing, which act as a molecular “switch”, resulting in severe PH-COPD [13]. Further research is needed to define if HIF-2 augmentation or suppression can be used as therapeutic targets in severe COPD or pulmonary vascular phenotype, respectively. 

## 5. Limitations 

There are several strengths and limitations in our systematic review and meta-analysis. We performed an exhaustive literature search to identify high-quality studies with strict quality assessment for inclusion. This is the first meta-analysis to include studies with severe PH-COPD using the latest hemodynamic definition obtained invasively via right heart catheterization and describe hemodynamic, clinical, and mortality outcomes with PH-specific therapy. Due to a lack of placebo-controlled RCTs, there is a significant gap in knowledge on the efficacy and safety of PH-specific therapy in severe PH-COPD. Only one of the studies included was an RCT, which limits the strength of the results. The severity of COPD was not reported in most studies, which can have a significant impact on clinically important outcomes such as the 6MWD and functional class, limiting the observed effect of pulmonary hypertension-specific therapy. In addition, there is a possibility of publication bias as unregistered studies or trials that are not published may have been excluded. Higher-quality data are required in the form of further RCTs to evaluate the therapeutic impact of PH-specific therapy in severe PH-COPD. 

## 6. Conclusions

This meta-analysis showed that treatment of severe PH-COPD with pulmonary vasodilator therapy resulted in a modest reduction in mPAP and PVR, and an increase in the cardiac index without a significant reduction in PaO_2_. In addition, there were fewer patients who remained in NYHA functional class III or IV with therapy; however, there was no significant difference in the 6MWD with treatment. Further studies in the form of placebo-controlled double-blinded RCTs are needed to investigate the use of PH-specific therapies in patients with severe PH-COPD or “pulmonary vascular phenotype” COPD.

## Figures and Tables

**Table 1:** Summary of quality assessment of cohort studies using Newcastle–Ottawa Scale (NOS).

NOS Criteria	Studies (Year)											
	Abuserewa, 2021 [22]	Brewis, 2015 [23]	Calcaianu, 2016 [24]	Faria-Urbina, 2018 [25]	Fossati, 2014 [26]	Girard, 2015 [27]	Hurdman, 2013 [28]	Jones, 1989 [29]	Lange, 2014 [30]	Madden, 2006 [31]	Tanabe, 2015 [32]	Wang, 2017 [33]
Representativeness of the exposed cohort		★		★	★	★	★	★	★	★	★	★
Selection of the nonexposed cohort	NA	NA	NA	NA	NA	NA	NA	NA	★	NA	NA	NA
Ascertainment of exposure	★	★	★	★	★	★	★	★	★	★	★	★
Demonstration that outcome of interest was not present at the start of the study	★	★	★	★	★	★	★	★	★	★	★	★
Comparability of cohort on the basis of the design or analysis	NA	NA	NA	NA	NA	NA	NA	NA		NA	NA	NA
Assessment of outcome	★	★	★	★	★	★	★	★	★	★	★	★
Follow-up duration >3 months	★	★	★	★	★	★	★		★	★	★	
Adequacy of follow-up of cohorts	★	★	★	★	★	★	★	★	★	★	★	★
Total (maximum of nine stars)	5	6	5	6	6	6	6	5	7	6	6	5

The methodological quality of retrospective or prospective observational studies was assessed using the Newcastle–Ottawa Scale (NOS) quality score. Each asterisk/star represents responses to the bias questionnaire. Each bias assessment part receives one star except comparability, which receives a maximum of 2 stars. Each star counts toward the total score. Score 7 represents a high quality. NA: not available or unable to extract.

**Table 2:** Summary of studies included in systematic review and meta-analysis.

Study ID	Title	Country	Study Design	Population Description	Treated/Total (*n*/*n*)	PH Specific Therapy Used (*n*)	Outcomes Assessed
Abuserewa 2021 [22]	Role of Selexipag in Chronic Obstructive Pulmonary Disease (COPD) Patients With Out-of-Proportion Pulmonary Hypertension	United States	Cohort study	PH-COPD patients treated with selexipag for “out-of-proportion” PH (FEV1 > 50%)	6	Selexipag	Hemodynamics, 6MWD, Borg scale
Brewis 2015 [23]	Severe pulmonary hypertension in lung disease: phenotypes and response to treatment	UK	Cohort study	Severe PH-COPD who received a minimum of 3 months of PH-targeted therapy.	71	ERA (16), prostanoid (4), combination (1)	NYHA, 6MWD, NT-proBNP, survival
Calcaianu 2016 [24]	Pulmonary Arterial Hypertension-Specific Drug Therapy in COPD Patients with Severe Pulmonary Hypertension and Mild-to-Moderate Airflow Limitation	France	Cohort study	Severe PH-COPD who were treated with PH-specific therapy.	28	ERA (23), PDE5 (1), Combination (2), other (2)	Hemodynamics, survival, NYHA class, BNP
Faria-Urbina 2018 [25]	Inhaled Treprostinil in Pulmonary Hypertension Associated with Lung Disease	United States	Cohort study	Severe PH patients evaluated at tertiary PH center; 22/72 patients had Group 3 PH.	8	Treprostinil	WHO-FC, echocardiography, 6MWD, SpO_2_
Fossati 2014 [26]	Long-term effect of vasodilator therapy in pulmonary hypertension due to COPD: a retrospective analysis	Switzerland	Cohort study	Retrospective review of patients seen at pulmonary hypertension clinic who had PH-COPD and received PH target therapy for at least 3 months.	27	Prostanoid (15), ERA (15), PDE5I (25)	Survival, NYHA, 6MWD, SpO_2_
Girard 2015 [27]	Severe pulmonary hypertension associated with COPD: hemodynamic improvement with specific therapy	France	Cohort study	Retrospective review of patients seen at pulmonary hypertension referral center who had PH-COPD.	26	ERA (11), PDE5I (11), Mix (3)	6MWD, NYHA, echocardiography, NT proBNP, SpO_2_
Hurdman 2013 [28]	Pulmonary hypertension in COPD: Results from the ASPIRE registry	UK	Cohort study	Consecutive patients seen at pulmonary hypertension referral center and diagnosed with PH-COPD were included, subdivided by severity of PH.	43/59	PDE-5I (31), ERA (10), Prostanoid (2)	Hemodynamics, survival, 6MWD, WHO-FC
Jones 1989 [29]	Pulmonary vasodilation with prostacyclin in primary and secondary pulmonary hypertension	UK	Nonrandomized experimental study	Twenty-three patients with PH underwent vasodilation testing with prostacyclin. Five patients had PH-COPD.	5	Prostacyclin IV	Hemodynamics
Lange 2014 [30]	Outcome of Patients with Severe PH due to Lung Disease with and without Targeted Therapy	Germany	Cohort study	Consecutive patients with a new diagnosis of Group 3 PH (retrospectively from a database and prospectively from a single center); 29/72 patients had PH-COPD, and 12 patients had severe PH-COPD.	12	Various, not reported	Hemodynamics, survival, 6MWD
Madden, 2006 [31]	A potential role for sildenafil in the management of pulmonary hypertension in patients with parenchymal lung disease	UK	Cohort study	Consecutive patients seen for Group 3 pulmonary hypertension at the referral center and treated with sildenafil; 4/7 patients had PH-COPD.	3	Sildenafil	Hemodynamics, 6MWD
Tanabe, 2015 [32]	Multi-institutional retrospective cohort study of patients with severe pulmonary hypertension associated with respiratory diseases	Japan	Cohort study	Patients with Group 3 severe PH; 18/70 patients had PH-COPD.	14/18	Beraprost (7), ERA (9), PDE5I (15)	Hemodynamics, survival
Vitulo, 2017 [19]	Sildenafil in severe pulmonary hypertension associated with chronic obstructive pulmonary disease: A randomized controlled multicenter clinical trial	Italy	Randomized controlled trial	Patients with COPD were screened for PH. Patients in whom PH was significantly out of proportion relative to COPD severity were included in the trial.	28	Sildenafil	Hemodynamics, BODE, 6MWD, QOL. PaO_2_
Wang, 2017 [33]	Hemodynamic and gas exchange effects of inhaled iloprost in patients with COPD and pulmonary hypertension	Australia	Nonrandomized experimental study	The efficacy and safety of iloprost inhalation were assessed in patients with COPD and PH; 37/67 patients had severe PH.	37	Iloprost	Hemodynamics, gas exchange parameters

6MWD: six-minute walk distance; BNP: brain-natriuretic peptide; BODE: body mass index, airflow obstruction, dyspnea, and exercise capacity; ERA: endothelin receptor antagonist; NYHA: New York Heart Association; PDE5I: phosphodiesterase type 5 inhibitor; QOL: quality of life questionnaire; SpO_2_: oxygen saturation; WHO-FC: World Health Organization functional class.

**Table 3:** Summary of transplant-free survival in COPD patients with severe PH treated with PH-specific therapy.

	Number of PH-COPD Patients (Treated Only)	Death + Transplant at End of Follow Up	Transplant-Free Survival at 1 Year	Transplant-Free Survival at 2 Years	Transplant-Free Survival at 3 Years
Brewis Emphysema 2015 [23]	31	23	74%	NA	32%
Brewis COPD 2015 [23]	40	24	82%	NA	50%
Calcaianu 2016 [24]	28	12	84%	63%	45%
Fossati 2014 [26]	27	12	92%	69%	54%
Hurdman 2013 [28]	43	Not reported	72%	38%	30%
Tanabe 2015 [32]	14	Not reported	Not reported	Not reported	37.5%
Total	183	NA	81%	57%	41%

## Data Availability

Data supporting this study is publicly available and referenced. No new data were created.
